# Death by Particles: The Link Between Air Pollution and Fatal Coronary Heart Disease in Women

**Published:** 2005-12

**Authors:** Rebecca Kessler

A growing body of evidence links chronic exposure to air pollution––especially particulate matter (PM)––with mortality resulting from a variety of heart, lung, and respiratory diseases. A new study corroborates this association, and indicates that women may be at greater risk than men of fatal coronary heart disease (CHD) as a result of exposure to airborne PM **[*EHP* 113:1723–1729]**. When ozone (O_3_) or sulfur dioxide (SO_2_) is also present, women’s risk appears even greater.

The study, by a team of epidemiologists at Loma Linda University, is part of the 22-year Adventist Health Study on the Health Effects of Smog. It followed 3,239 nonsmoking, non-Hispanic white adults in several mainly urban areas in California from 1976 to 1998. The researchers associated CHD deaths with prior exposure to various levels of several common air pollutants: PM_2.5_, PM_10–2.5_, PM_10_, O_3_, SO_2_, and nitrogen dioxide (NO_2_).

Participants completed a baseline health and lifestyle questionnaire in 1976, and four subsequent questionnaires covering personal sources of air pollution, such as secondhand tobacco smoke and fumes in the workplace. The researchers used airport visibility measurements (for PM_2.5_ only) and data from state-run air pollution monitors (for all other pollutants) to estimate pollutant levels over time for the zip code centroids of participants’ work sites and residences. Documented pollutant levels ranged from negligible to above legal limits. California’s death certificate files and the National Death Index provided data on numbers and causes of deaths.

The researchers found that CHD caused 23.7% of all the deaths in the study cohort (155 women and 95 men). Adjusting for past smoking, body mass index, education level, frequency of eating meat, and calendar year (as PM levels declined over the study period), the researchers conducted statistical analyses to determine whether fatal CHD was associated with long-term exposure to the pollutants, either singly or in combinations of single gases and PM.

Women showed a relative risk for fatal CHD of 1.42, 1.38, and 1.22 with each increase of 10 micrograms per cubic meter (μg/m^3^) of airborne PM_2.5_, PM_10–2.5_, and PM_10_, respectively, in the air pollution they encountered during the four years preceding death. Postmenopausal women showed higher relative risks of 1.49, 1.61, and 1.30 for each 10 μg/m^3^ increase in PM_2.5_, PM_10–2.5_, and PM_10_, respectively. Neither O_3_, SO_2_, nor NO_2_ was associated with fatal CHD on its own. O_3_ and to a lesser degree SO_2_ (but not NO_2_) increased the effect of all sizes of PM. O_3_ in conjunction with PM_2.5_ yielded the most striking results: a relative risk of 2.0 in all women. Contrary to findings from several other studies that found increased risk of cardiopulmonary deaths due to PM in both genders, men showed no response to any of the pollutants.

The researchers highlight several physiological mechanisms that may explain their findings. Short-term exposure to PM is known to increase arrhythmia, inflammation, and blood viscosity, and to decrease heart rate variability, among other adverse effects that could lead to fatal CHD. Other findings show that O_3_ exposure increases lung permeability, perhaps easing PM’s entry into the bloodstream. Finally, several studies have indicated that PM deposits differently—and perhaps more harmfully—in women’s lungs than in men’s. This may provide a starting point for teasing out the study’s finding of an association between PM and risk of fatal CHD in women, but not in men.

